# Comparison of nine tractography algorithms for detecting abnormal structural brain networks in Alzheimer’s disease

**DOI:** 10.3389/fnagi.2015.00048

**Published:** 2015-04-14

**Authors:** Liang Zhan, Jiayu Zhou, Yalin Wang, Yan Jin, Neda Jahanshad, Gautam Prasad, Talia M. Nir, Cassandra D. Leonardo, Jieping Ye, Paul M. Thompson, for the Alzheimer’s Disease Neuroimaging Initiative

**Affiliations:** ^1^Imaging Genetics Center, University of Southern California, Los AngelesCA, USA; ^2^Department of Neurology, Psychiatry, Pediatrics, Engineering, Radiology, and Ophthalmology, Keck School of Medicine, University of Southern California, Los AngelesCA, USA; ^3^School of Computing, Informatics, and Decision Systems Engineering, Arizona State University, TempeAZ, USA; ^4^Center for Evolutionary Medicine and Informatics, The Biodesign Institute, Arizona State University, TempeAZ, USA

**Keywords:** Alzheimer’s disease, brain network, tractography, classification, PCA, GLRAM, diffusion MRI

## Abstract

Alzheimer’s disease (AD) involves a gradual breakdown of brain connectivity, and network analyses offer a promising new approach to track and understand disease progression. Even so, our ability to detect degenerative changes in brain networks depends on the methods used. Here we compared several tractography and feature extraction methods to see which ones gave best diagnostic classification for 202 people with AD, mild cognitive impairment or normal cognition, scanned with 41-gradient diffusion-weighted magnetic resonance imaging as part of the Alzheimer’s Disease Neuroimaging Initiative (ADNI) project. We computed brain networks based on whole brain tractography with nine different methods – four of them tensor-based deterministic (FACT, RK2, SL, and TL), two orientation distribution function (ODF)-based deterministic (FACT, RK2), two ODF-based probabilistic approaches (Hough and PICo), and one “ball-and-stick” approach (Probtrackx). Brain networks derived from different tractography algorithms did not differ in terms of classification performance on ADNI, but performing principal components analysis on networks helped classification in some cases. Small differences may still be detectable in a truly vast cohort, but these experiments help assess the relative advantages of different tractography algorithms, and different post-processing choices, when used for classification.

## Introduction

Alzheimer’s disease (AD) – the commonest form of dementia – is characterized by memory loss in its early stages, typically followed by a progressive decline in other cognitive domains (Braak and Braak, 1996; Bartzokis, 2011; Braskie et al., 2011; Hua et al., 2013). Recent models of AD suggest that cognitive deficits arise from the progressive disconnection of cortical and subcortical regions, involving neuronal loss and white matter (WM) injury (Delbeuck et al., 2003). Several magnetic resonance imaging (MRI) analysis methods can track structural atrophy. Diffusion-weighted MRI (DWI), a variant of standard anatomical MRI, is sensitive to microscopic WM injury not always detectable with standard anatomical MRI. DWI tracks anisotropic water diffusion along axons, revealing microstructural WM fiber bundles connecting cortical and subcortical regions. With whole-brain tractography, one can reconstruct major fiber bundles in the brain’s anatomical network (LeBihan, 1990). In both AD and mild cognitive impairment (MCI), diffusion MRI studies have associated cognitive impairment with progressive deterioration in the corpus callosum, cingulum, superior longitudinal fasciculus, and fornix (Medina et al., 2006; Stebbins and Murphy, 2009; Liu et al., 2011; Daianu et al., 2013; Nir et al., 2013; Jin et al., 2015).

Many studies of brain disease – not just AD – use whole-brain tractography to assess large-scale connections in the brain. Tractography is a method used to reconstruct the pathways of major WM fiber bundles, by fitting a curved path through the directional diffusion data at each voxel. Tractography can reveal brain abnormalities in multiple sclerosis (Mesaros et al., 2012), a variety of cognitive disorders (Catani, 2006), Parkinson’s disease (García-Gomar et al., 2013), brain trauma (Dennis et al., 2015b), psychiatric conditions such as body dysmorphic disorder (Arienzo et al., 2013), and even in genetics (Jin et al., 2011, 2013). To assess these differences, dozens of tractography algorithms have been developed (Behrens et al., 2007; Kreher et al., 2008; Fillard et al., 2009) but there is little consensus on which method is the best to use, and this may also depend on the goal of the study. To the best of our knowledge, no empirical studies have compared tractography methods for studies of brain disease.

There are two main steps in the tractography: the first is to fit a diffusion model at each voxel of the image, and the second is fiber tracking across voxels. The most straightforward way is to use the standard tensor model, which requires at least six diffusion-weighted images (DWIs) and one baseline (non-diffusion-weighted) image to estimate the six unknown parameters of the tensor. The local dominant fiber direction at each voxel is then estimated as the eigenvector associated with the largest eigenvalue of the tensor. Fibers can be followed across voxels using greedy algorithms such as “fiber assignment by continuous tracking” (FACT), which builds streamlines^1^ across the image by following the diffusion tensors’ principal eigenvectors in the current direction of propagation (Mori et al., 1999). More complex models, based on multiple tensors, orientation distribution functions (ODFs), or “ball-and-stick” models, can extract multiple fiber directions per voxel.

The two main classes of fiber tracking methods are deterministic and probabilistic approach. Deterministic methods tend to be simple and fast. One of the first deterministic tractography approaches, FACT, can be run on a 3D brain image in several minutes. Probabilistic algorithms may require longer computation times. On a single processor, it typically takes several hours to run the Hough method (Aganj et al., 2011), one of the probabilistic algorithms, which fits vast numbers of polynomial curves through an ODF and tends to find the most dominant tracts of the brain using global optimization. Clearly, different algorithms recover different sets of fibers (Zhan et al., 2013b; Dennis et al., 2015a), and the fiber bundles that best differentiate patients from controls may be extracted by some algorithms but not others. When applied to detect brain differences in disease, it is not clear how tractography algorithms differ or if some are more sensitive to differences than others. This depends on which fibers are extracted and how accurately, the level of extraneous fibers and noise, and whether the fiber bundles affected most by the disease are extracted or missed.

Some prior studies compared different tractography algorithms for accuracy and robustness to image noise (Lazar and Alexander, 2003; Huang et al., 2004; Moldrich et al., 2010; Fillard et al., 2011). In Lazar and Alexander (2003), a Monte Carlo simulation was used to study the influence of principal direction estimation and streamline integration methods on the robustness to noise of DTI tractography. Fillard et al. (2011) used a hardware phantom to compare 10 fiber reconstruction methods at different signal-to-noise (SNR) levels. Phantom-based evaluations use simple tract shapes, which tend to be more regular than tracts in the living brain. Huang et al. (2004) created several noisy versions of a high-SNR mouse brain dataset to compare fiber bundle selection strategies. Moldrich et al. (2010) tested several tractography algorithms for studying WM pathways in the *ex vivo* mouse brain. Using deterministic and probabilistic algorithms across a range of regions of interest, they found that probabilistic tractography was more robust than deterministic tractography for visualizing both white and gray matter (GM) pathways. Moreover, as far as we know, no empirical studies have compared tractography methods for studies of brain disease.

Based on tractography, there at least two common ways that the integrity and connectivity of WM can be studied, i.e., the analysis of individual anatomically meaningful tracts (Jin et al., 2012, 2014) and the connectivity strength between parcellated GM regions (Zhan et al., 2012a,b, 2015). In the latter, brain connectivity maps (structural networks) are often computed by combining the tracts with an anatomical parcellation scheme (Jahanshad et al., 2011; Zhan et al., 2013b). Before further analysis, the brain networks can be thresholded, to keep only those connections with a weight or value, for example nodal degree (number of connections), higher than a given threshold. Network analysis of these brain connectivity matrices can reveal organizational properties of brain networks. Clearly, the tractography and feature extraction method choices affect how well we can identify disease-related differences. To understand how these choices affect how well we can detect effects of AD, we applied nine tractography methods to the Alzheimer’s Disease Neuroimaging Initiative (ADNI) dataset, and investigated five feature extraction approaches on the computed networks. Our goal was to evaluate the performance of different tractography and feature extraction methods. Rather than test their anatomical accuracy, we focused on their ability to detect disease-related effects.

## Materials and Methods

### Subject Demographics and Image Acquisition

Data used in preparing this article were obtained from ADNI2, the second stage of the Northern American ADNI^2^. ADNI’s primary goal is to test whether serial MRI, positron emission tomography (PET), other biological markers, and clinical and neuropsychological assessments can be combined to measure the progression of MCI and early AD. For up-to-date information, please see http://www.adni-info.org.

In our experiments, we analyzed 202 subjects’ diffusion MRI and structural MRI data collected from 16 sites across the United States and Canada for the ADNI2 project. Note that only around one third of the ADNI2 participants have diffusion MRI scans, and the other two-thirds are scanned with resting state functional MRI or arterial spin labeling (ASL). Detailed inclusion and exclusion criteria are found in the ADNI2 protocol^3^. Subjects are divided into three broad diagnostic categories: normal elderly controls (NCs), people with MCI and patients with AD. Subject demographics are summarized in **Table 1**.

**Table 1:** Summary of ADNI data used in this study.

	Normal control (NC)	MCI (MCI)	AD	Total
Number	51	112	39	202
Age (y)	69.69 ± 15.43	71.68 ± 9.89	75.56 ± 9.11	71.92 ± 11.54
Sex	29F	41F	14F	84F

There is no age difference among these groups based on a one-way ANOVA (*p* = 0.0536) but the proportion of women in HC group (56.86%) was higher than that of the AD (35.90%) or MCI groups (36.61%).

Each subject underwent whole-brain MRI scanning on 3-Tesla GE Medical Systems scanners. T1-weighted SPGR (spoiled gradient echo) sequences (256 x 256 matrix; voxel size = 1.2 × 1.0 × 1.0 mm^3^; TI = 400 ms; TR = 6.98 ms; TE = 2.85 ms; flip angle = 11°), were collected as well as DWI (128 × 128 matrix; voxel size: 2.7 × 2.7 × 2.7 mm^3^; scan time = 9 min; more imaging details may be found at http://adni.loni.usc.edu/wp-content/uploads/2010/05/ADNI2_GE_3T_22.0_T2.pdf). 46 separate images were acquired for each DWI scan: five T2-weighted images with no diffusion sensitization (*b_0_* images) and 41 DWIs (*b* = 1000 s/mm^2^). The DWI protocol for ADNI was chosen after a detailed evaluation of different protocols that could be performed in a reasonable amount of time; we reported these comparisons previously (Jahanshad et al., 2010; Zhan et al., 2013a). All T1-weighted MR and DWI images were checked visually for quality assurance to exclude scans with excessive motion and/or artifacts; all scans were included.

### Comparing Tractography Methods

**FIGURE 1:**
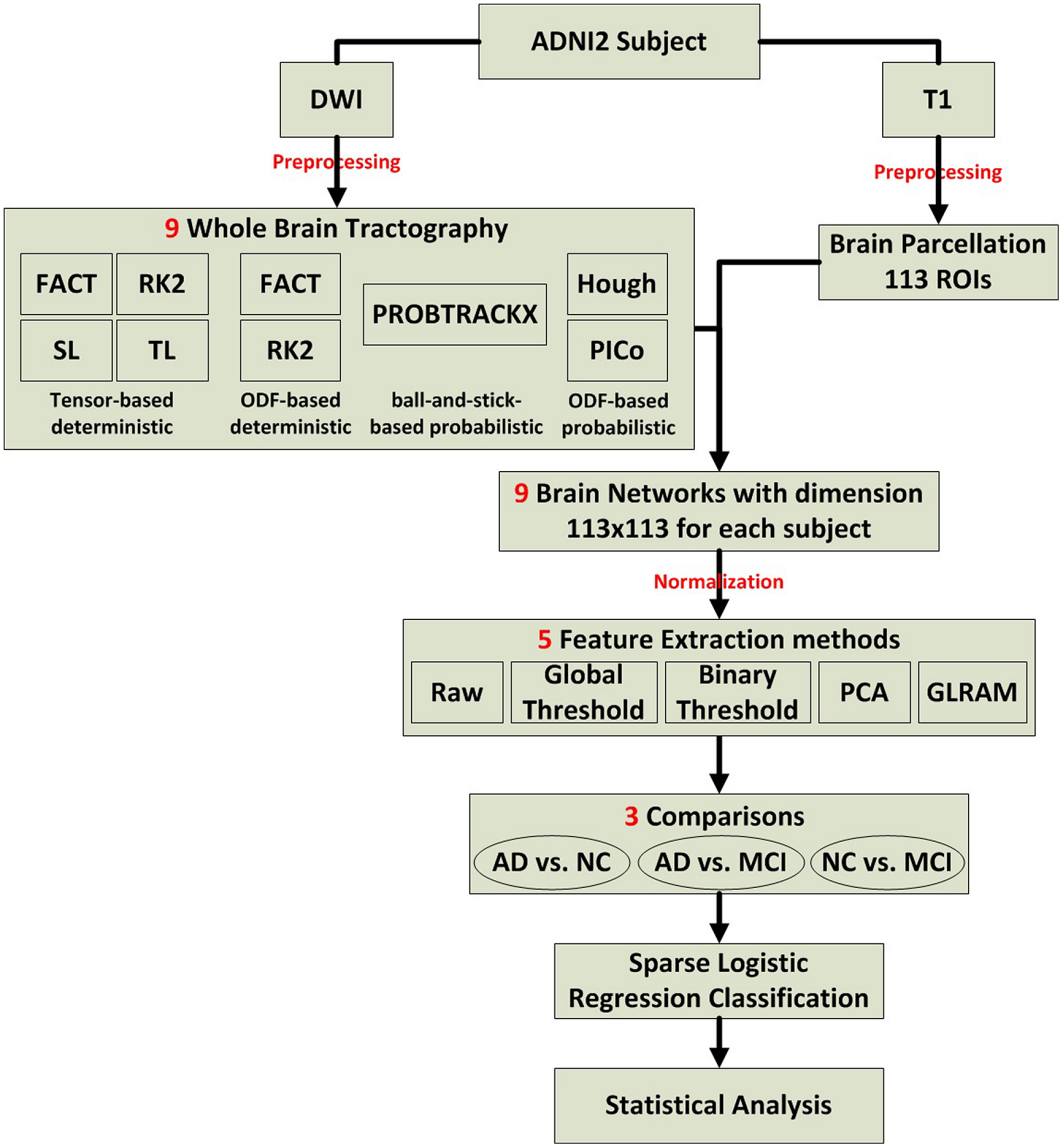
**Flow chart describing the steps taken in this study to create, analyze, and compare structural networks**.

We evaluated several tractography and feature extraction choices, and how well they detect disease effects in ADNI2. **Figure 1** illustrates the overall study design, which is detailed in Section “Preprocessing to Statistical Analysis.”

### Preprocessing

For each subject, extra-cerebral tissue was removed from the T1-weighted anatomical scans using ROBEX, a robust automated brain extraction program trained on manually “skull-stripped” MRI data (Iglesias et al., 2011). Skull-stripped volumes were visually inspected, and manually edited if needed. Anatomical scans then underwent intensity inhomogeneity normalization using the MNI *nu_correct* tool^4^. To align data from different subjects into the same 3D coordinate space, each anatomical image was linearly aligned to a standard brain template (the Colin27; Holmes et al., 1998) using FSL *flirt* (Jenkinson et al., 2002).

Then each subject’s raw DWI volumes were aligned to the *b_0_* image using the FSL *eddy-correct* tool^5^ to correct for head motion and eddy current distortions. The gradient table was also corrected accordingly. Non-brain tissue was removed from the DWIs using the Brain Extraction Tool (BET) from FSL (Smith, 2002). To correct for echo-planar induced (EPI) susceptibility artifacts, which can cause distortions at tissue-fluid interfaces, skull-stripped *b_0_* images were linearly aligned and then elastically registered to their respective preprocessed T1-weighted structural scans using an inverse consistent registration algorithm with a mutual information cost function (Leow et al., 2007). The resulting 3D deformation fields were then applied to the remaining 41 DWI volumes to generate full preprocessed DWI dataset for the downstream computation.

### Whole Brain Tractography

Nine different tractography methods were evaluated, including deterministic and probabilistic approaches. Among the deterministic methods were four tensor-based deterministic algorithms: FACT (Mori et al., 1999), the second-order Runge–Kutta (RK2) method (Basser et al., 2000), the tensorline (TL; Lazar et al., 2003) and interpolated streamline (SL) methods (Conturo et al., 1999) and two deterministic tractography algorithms based on fourth order spherical harmonic derived ODFs – FACT and RK2. We also tested three probabilistic approaches: one was “ball-and-stick model based probabilistic tracking” (Probtrackx) from the FSL toolbox (Behrens et al., 2007) and the other two were based on ODFs represented by fourth order spherical harmonic series: the Hough voting method (Aganj et al., 2011) and the probabilistic index of connectivity (PICo) method (Parker et al., 2003).

Developed in the 1990s, FACT was one of the first deterministic tractography methods for DTI. It is still perhaps the most popular fiber tracking approach for both scientific and clinical applications. The main idea is to trace out the path of the diffusion tensor’s principal eigenvector (the unit eigenvector associated with the maximum eigenvalue) in the image, while testing for sudden transitions in the local fiber orientation. The RK2 algorithm uses the second order Runge–Kutta method to solve a differential equation to more reliably estimate the fiber trajectory. The key idea behind RK2 is to equate the tangent vector with the principal eigenvector. The TL method tracks fibers using diffusion tensor “deflection,” and uses the entire tensor to determine the direction of tract propagation, instead of just the principal eigenvector. The SL method reconstructs fiber trajectories throughout the brain by tracking the direction of greatest diffusion in interpolated steps (typically 0.5 mm). All six deterministic tracking approaches (tensor-FACT, RK2, SL, TL, and ODF-FACT, RK2) were applied using their implementations in the Diffusion Toolkit^6^. Fiber tracking was restricted to regions where fractional anisotropy (FA) ≥ 0.2 to avoid GM and cerebrospinal fluid; fiber paths were stopped if the fiber direction encountered a sharp turn (with a critical angle threshold ≥ 30°). Sharp “right-angle” turns may be biologically possible in some cases (Wedeen et al., 2012), but allowing right-angle turns in tractography would create large numbers of false positive pathways at fiber crossings. Usually deterministic approaches generate around 30,000–50,000 non-duplicated fibers (3D curves) per brain.

Probtrackx was performed after Bedpostx has been applied. Bedpostx stands for Bayesian Estimation of Diffusion Parameters Obtained using Sampling Techniques (Behrens et al., 2007). The *X* stands for modeling crossing fibers. Bedpostx runs Markov Chain Monte Carlo sampling to build up distributions on diffusion parameters at each voxel. It creates all the files necessary for running probabilistic tractography. In our study, up to three fibers were modeled per voxel. Once Bedpostx had been run, we chose all voxels with FA ≥ 0.2 as the seeds. Following Bedpostx, Probtrackx was run on each individual seed voxel. Probtrackx repeatedly samples from the voxel-wise principal diffusion direction calculated in Bedpostx, creating a new streamline at each iteration. This builds a distribution on the likely tract location and path, given the data. 1000 iterations were run to ensure convergence of the Markov chains, from which the posterior distributions of the local estimate of the fiber orientation distribution were sampled.

Hough probabilistic tractography was performed with code provided by the authors (Aganj et al., 2011). In short, ODFs at each voxel were computed using the normalized and dimensionless constant solid angle ODF estimator, derived for Q-ball imaging (QBI) as in (Aganj et al., 2010). Tractography was performed by probabilistically seeding voxels with a prior probability based on the FA value (FA ≥ 0.2). All possible curves passing through a seed point were estimated and each received a score estimating the probability of the existence of the fiber, computed from the ODFs. Then the Hough transform voting process was adopted to determine the best fitting curves through each point. Each subject’s dataset contained approximately 10,000 non-duplicated fibers per brain. Hough probabilistic tractography aims to optimize the fiber pathway globally, so there is no explicit upper limit on the number of detectable crossing fibers although the data angular resolution will limit this in practice.

Probabilistic index of connectivity probabilistic tractography was conducted with Camino^7^. Seed points were chosen at those voxels with FA ≥ 0.2. ODFs were estimated using fourth order Spherical Harmonics and a maximum of three local ODF maxima (where fibers mix or cross) were set to be detected at each voxel. Then, a probability density function (PDF) profile can be produced from the derived local ODF maxima. Monte-Carlo simulation was used to generate fibers emanating from seed points inside the entire brain. Streamline fiber tracking followed the voxel-wise PDF profile with the Euler interpolation method, for 10 iterations per each seed point. The maximum fiber turning angle was set to 30°/voxel. Tracing stopped at any voxel whose FA was less than 0.2. This approach generates many more fibers than other methods used in this study.

The parameter settings for each of these tractography algorithms can also be varied, leading to a huge number of comparisons. To avoid that, we used parameter settings for each method that had been previously optimized by our group or others; in most cases, they were the default parameter settings of the methods. To avoid undue complexity, we concede that changing these parameters could conceivably affect how the methods are ranked. Moreover, all fibers shorter than 10 mm were filtered out, as these were much more likely to be false positive fibers.

### Computing Brain Networks

One hundred thirteen cortical and subcortical ROIs (listed in the Supplement) were defined using the Harvard Oxford Cortical and Subcortical probabilistic atlas (Desikan et al., 2006). Midline cortical masks were bisected into left and right components, to define separate hemispheric ROIs for each cortical region. Since this is a probabilistic atlas, masks were thresholded at 10% to ensure inclusion of tissue along the gray-WM interface, where fiber orientation mapping and tractography are most reliable. To register these ROIs to each subject’s DWI space, we used FSL’s *flirt* function to determine the optimal affine transformation between the MNI152 T1 average brain (on which the Harvard Oxford probabilistic atlases are based) and each subject’s skull-stripped T1-weighted image (see Preprocessing), as well as the optimal affine transformation between each subject’s skull-stripped T1 and unique FA images. We used a 12 degree-of-freedom registration with a mutual information cost function. After combining the above two steps’ transformation matrices, we transformed the 113 ROIs to each subject’s DWI space using nearest-neighbor interpolation. To ensure that ROI masks did not overlap with each other after registration, each voxel was uniquely assigned to the mask for which it had the highest probability of membership. We admit there are other ways to define ROIs, such as FreeSurfer parcellation or using non-linear registration method with other atlases. However, testing all possible paracellations was beyond the scope of this paper, here we used the same brain parcellation scheme to compare different tractography methods.

For each ROI pair, the number of detected fibers connecting them was determined from the tractography results in Section “Whole Brain Tractography.” A fiber was considered to connect two ROIs if it intersected both of them. This process was repeated for all ROI pairs, to compute a whole brain fiber connectivity matrix. This matrix is symmetric, by definition, and has a zero diagonal (no self-connections; Zhan et al., 2013b). To avoid computation bias in the later feature extraction and evaluation sections, we normalized each brain matrix by the maximum value in the matrix, as matrices derived from different tractography methods have different scales and ranges.

### Feature Extractions

Up to this step, each subject has nine matrices. Now we need to select feature extraction methods for the classification. Typically the training of a classifier requires the subjects to be described by feature vectors. Therefore the brain networks, represented by matrices – equivalent to very high-dimensional vectors – cannot easily be used to train classifiers, without some dimension reduction or feature selection. As such a feature extraction process is needed to extract useful information from brain networks and represent it in a simpler vector form. One simple approach is to directly use all of the numerical values from the matrix representing the brain network. We call this approach the “raw features” approach, where we just use all the numerical information in the network. One apparent advantage is that this approach requires no extra computation. Even so, the approach generates a huge feature space. Also, the network matrix elements are always corrupted by a certain level of noise. With the huge feature space and limited sample sizes, classifiers may be especially susceptible to such noise. To make the learning process more robust, we chose some commonly used feature selection techniques by thresholding the matrices globally or at individual level, to reduce the number of features. We also chose to use one of the mostly used dimension reduction methods, principal components analysis (PCA) to reduce the number of features. We also tested another closely related approach for dimension reduction on the 2D matrices, based on generalized low-rank approximation of matrices (GLRAM). Thus, for each matrix belonging to the 202 subjects, five different feature extraction methods were selected and described as follows:

(1) Raw features. In our study, the matrices are always symmetric, so we used all the values in the upper triangle of the matrices as features for classification. This feature space is a 6328 × *N* vector: 6328 = (113 × 112/2) and *N* is the number of subjects.
(2) Global threshold. In this method, we first compute the mean matrix by averaging the matrices across all subjects. We then rank the elements of the upper triangle of the mean matrix, and record the locations of the largest 5–40% elements, respectively. (We experimented with a wider range from 5 to 100% but obtained best results with the largest 5–40% of the elements, so we report experimental performance in this range.) We then used this subset of elements from the subject matrices as features for classification. In other words, if the threshold = 0.25, we first computed a mean network by averaging all subjects’ networks, then masked the top 25% elements’ positions in the upper triangle of the mean network. Based on this “global” mask, all values except in these masked positions were set to zero for each subject’s network. Then the upper triangle of the thresholded network was defined as features for classification. The feature space is a 6328x*N* vector with all values in unmasked positions set to zero and *N* is the number of subjects.
(3) Individual binary threshold. In this method, we convert the values in the matrices into binary variables. If a value exceeds a given threshold, it is set to 1, and 0 otherwise. This individual threshold is obtained by ranking the elements within each subject, and a value is set to 0/1 depending on its relative ranking among all entries within that same matrix. We vary the threshold from the top 5% to top 40% (to be consistent with global threshold method), and perform classification at each thresholding level. The feature space is a 6328x*N* vector, but with all values less than threshold set to zero. Again, *N* is the number of subjects.
(4) PCA. The raw feature space is large (113 ^∗^ 112 /2 = 6328 matrix elements or features), and in the training phase we have fewer than 100 samples. To tackle the ‘large dimension, small sample size’ problem, we employed PCA to reduce the data dimensionality. We first take the upper diagonal as features, and form a sample-by-feature input matrix. We then perform PCA on the input matrix to perform dimension reduction by keeping the first *k* principal components, where we vary *k* from 10 to 150. The reduced input matrix is then used to perform classification. The feature space is a *k* × *N* vector, where *k* is the parameter we investigated in Section “Comparing Classification Performance after Using GLRAM,” which is 10–150. Again, *N* is the number of subjects.
(5) GLRAM (Ye, 2005). One way to reduce the matrix dimension is to use a generalized low-rank approximation, in which we collectively factorize all the subject matrices into three components. That is, for the matrix of each subject M_i_, we factorize it as M_i_ = L × X_i_ × R, where L ∈ R^d×k^ and R ∈ R^k×d^ are shared orthonormal transformations for all matrices, and X_i_ ∈ R^k×k^ is a reduced matrix. We use X_i_ as the new feature representation for classification. One important parameter in GLRAM is the reduced row/column dimensionality. Again, a range of parameter values was investigated to seek the “best” option for the classification. The feature space is am *m^2^* × *N* vector, *N* is the number of subjects, and *m* is the reduced matrix dimension.

### Classifications

After feature extraction from the matrices, we evaluated these feature vectors for three between-group comparisons including AD vs. NC, AD vs. MCI, NC vs. MCI. One could define some metric of group separation to summarize the results, but in practice algorithms may have different strengths and weaknesses depending on the groups compared, as different sets of fibers may differ across diagnostic groups. In these comparisons, AD, MCI, and NC are groups listed in **Table 1**. We performed the classification as follows:

(1) We selected all the subjects relevant to the classification task.
(2) We performed *z*-score normalization for each feature, i.e., for each feature, we subtract the mean value of the feature across the selected subjects and divide by the standard deviation.
(3) The class labels are typically unbalanced in our study. To avoid bias, we constructed 20 balanced training/testing sample splits, as follows:
  (a) Randomly draw 85% of the data from the smaller class for training, and the remaining 15% for testing.
  (b) In the larger class, we match the same number of training samples by a random subsampling, and the rest are put in the test set.
  For example, in the AD/NC task, we have 39 AD samples and 51 NC samples. We first use 33 AD samples (85%) for training and six AD samples (15%) for testing. Then we randomly select 33 NC samples and include them in the training set and include the remaining 18 samples in the test set.
(4) In each training/testing split, we use the training set to train a sparse logistic regression classifier (the classifier parameters are estimated from the training data via fivefold cross validation), and we test the classifier’s effectiveness on the test set. The AUC (area under ROC curve) is computed by averaging the trapezoidal approximations for the curve created by true positive rate (TPR) and false positive rate (FPR). TPR is the proportion of positive samples correctly identified as positive and FPR is the proportion of negative samples correctly identified as negative. Multiple classification models were generated for every cross fold. Each provides a prediction, positive or negative, for the given class instance. In the machine learning field, AUC is a common measure of classification performance. For tractography, other measures may also be useful, such as ground truth accuracy and completeness of fiber recovery. So admittedly we are only studying one metric, although it is probably as important as the others.

### Statistical Analysis

However, it may be inaccurate to characterize the tractography algorithm performance using the averaged AUC based on only 20 splits. Thus, the 95% confidence interval (CI) for the AUC was computed and one-way analysis of variance (ANOVA) was performed on the AUCs. Our null hypothesis (*H_0_*) was: there is no significant difference in the AUCs from different tractography algorithms. If *H_0_* was rejected, *post hoc* multiple group comparisons were planned to investigate where these differences are. All statistical analysis steps were performed using IBM SPSS Statistics V22. The experiment-wise alpha threshold was set to *p* < 0.05. In our study, a Bonferroni correction was adopted for multiple hypothesis correction. However, all the *p*-values reported here have been adjusted by SPSS with the appropriate correction for the effective number of multiple comparisons used. For instance, for a three-group experiment, a pairwise comparison (i.e., a *t*-test) that yields a *p-*value of 0.016 would be considered significant at the 0.05 level, because 0.016 < (0.05/3). Instead of giving the nominal two-tailed *p*-value, SPSS adjusts the *p*-value by multiplying it by 3, in this case giving a Bonferroni *p* of 0.048 (0.016 times 3). (SPSS adjusts the actual *p-*value by applying the Bonferroni correction backward.)

## Results

### Comparing Brain Networks

**Table 2** compares fiber lengths and numbers for different tractography algorithms. We did not report them for Probtrackx, which does not output these parameters. In **Table 2**, ODF-based deterministic tractography (ODF-FACT, RK2) tended to generate more false positive fibers (such as shorter fibers) than tensor-based deterministic tractography (tensor-FACT, RK2, SL, and TL). Moreover, the “noisy fiber ratio” (see last column in **Table 2**) suggests that deterministic approaches (tensor-FACT, RK2, SL, TL, and ODF-FACT, RK2) and PICo may generate more false positive fibers than Hough. Hough fits fibers by global optimization, but greedy algorithms process small neighborhoods at each step, much like FACT or PICo. Clearly, the differences in fiber numbers and lengths may affect downstream analysis, as described below.

**Table 2:** Compares the fiber length and number for different tractography algorithms except Probtrackx.

		Fiber length (mm)			Total number		
		Mean	Median	Maximum	Fibers	Short fibers	Ratio (%)
Tensor	FACT	33.30 ± 2.85	22.55 ± 2.09	229.48 ± 52.81	43301 ± 7359	11206 ± 1466	25.88
	RK2	38.22 ± 3.37	25.75 ± 2.72	279.67 ± 48.05	47699 ± 7808	12073 ± 1465	25.31
	SL	42.95 ± 3.90	31.36 ± 3.42	291.38 ± 68.39	48205 ± 7853	9756 ± 1168	20.24
	TL	43.65 ± 3.99	32.44 ± 3.57	291.12 ± 43.07	44489 ± 7526	8366 ± 1031	18.80
ODF	FACT	23.87 ± 1.76	15.80 ± 1.18	152.29 ± 14.81	56674 ± 10063	20051 ± 2984	35.38
	RK2	27.03 ± 2.18	17.36 ± 1.53	188.16 ± 29.66	65626 ± 11202	22884 ± 3184	34.87
ODF	PICo	26.37 ± 1.97	17.92 ± 1.22	174.58 ± 21.70	503527 ± 82907	144907 ± 19699	28.78
	Hough	54.51 ± 3.29	57.12 ± 0.55	109.49 ± 1.96	10000	374 ± 309	3.74

Values are computed from all 202 subjects; mean and median values of fiber length are computed using all fibers. We noticed most short fibers were extracted at the brain periphery, mainly due to noise in the images, and hence, all fibers shorter than 10 mm were called “short fibers” and were removed from downstream analysis. Column Ratio is the proportion of all fibers that were short fibers.

**FIGURE 2:**
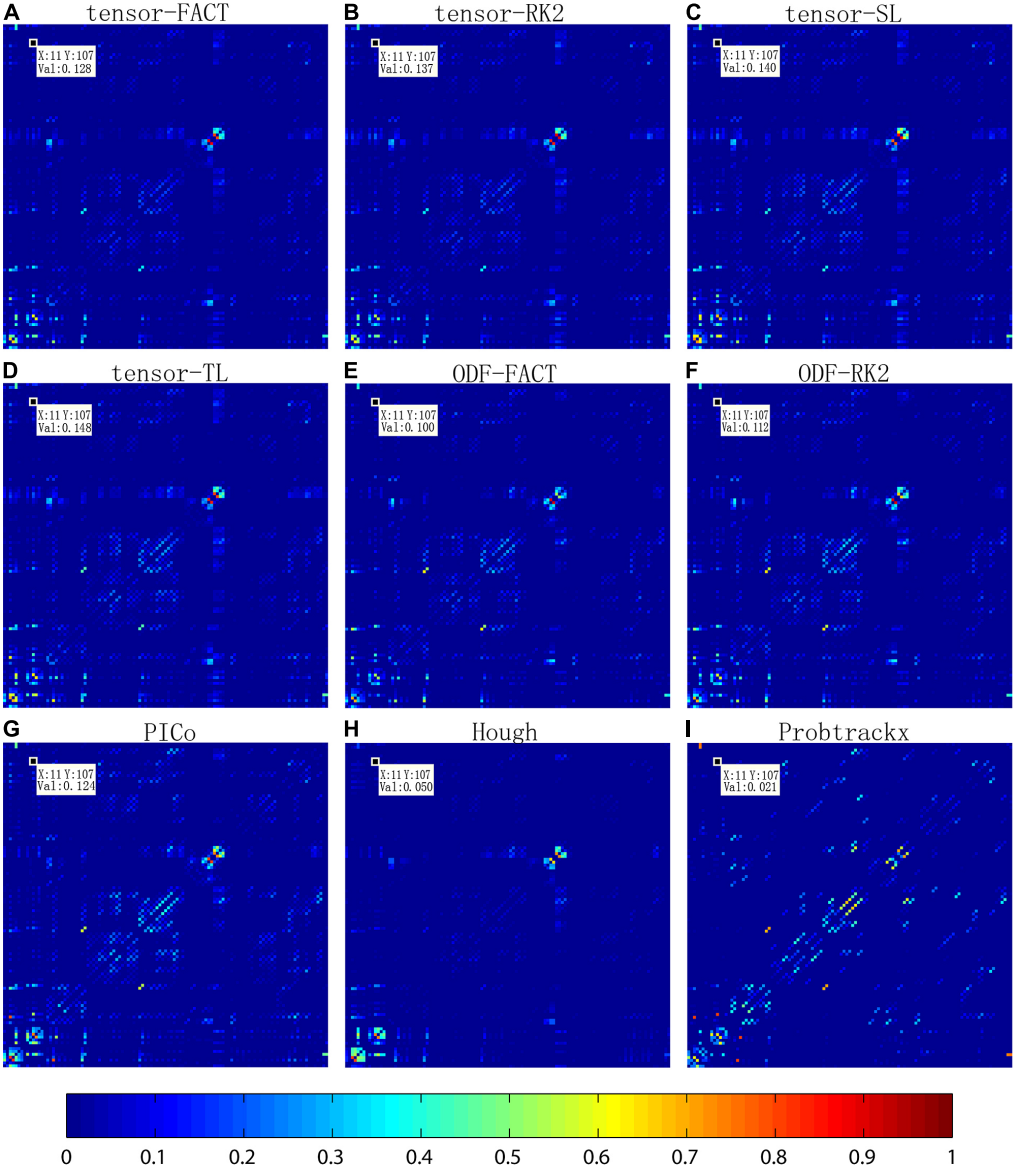
**Comparison of averaged normalized brain networks from nine different tractography algorithms, including **(A)** tensor-based FACT; **(B)** tensor-based RK2; **(C)** tensor-based SL; **(D)** tensor-based TL; **(E)** ODF-based FACT; **(F)** ODF-based RK2; **(G)** ODF-based PICo; **(H)** ODF-based Hough, and **(I)** Ball-and-stick model based Probtrackx, from all 202 subjects.** In each network, each cell represents the connectivity between each pair of ROIs; the ROI index runs from 1 to 113 from left to right and from bottom to top. ROI names are detailed in the Supplement. Visually, brain networks from different tractography algorithms may have similar patterns but in reality, the recovered brain network varies, as shown by the value in the randomly selected cell (11,107).

**Figure 2** compares the mean normalized brain networks for nine tractography algorithms. This mean normalized network is computed from all 202 subjects. Visually, the deterministic approaches – including tensor-FACT, RK2, SL, TL, and ODF-FACT, RK2 – have very similar connection patterns and the probabilistic approaches (PICo, Hough, and Probtrackx) have very different patterns. The apparent connectivity values are also different. If we randomly consider one connection between the 11th ROI and the 107th ROI (see Supplement for the numbering of ROIs), the apparent connectivity varies from a minimum of 0.021 (Hough) and maximum of 0.148 (tensor-TL).

### Comparing Classifications Based on Raw Matrices

**FIGURE 3:**
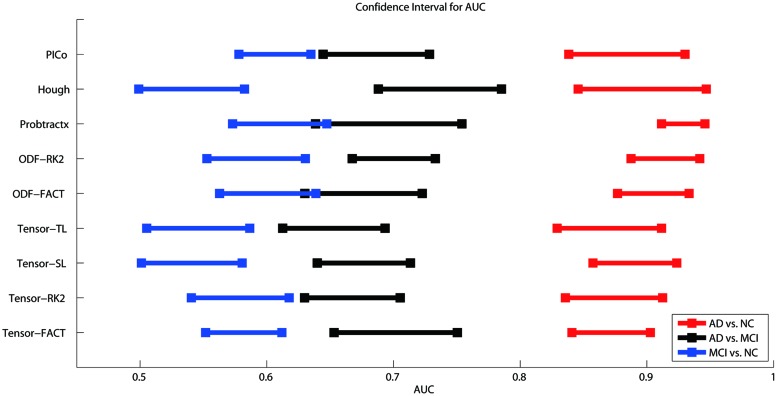
**Ninety five percent confidence intervals (CI) for the AUC (classification accuracy) for three diagnostic tasks represented by the three color bars (blue, black, and red) for nine tractography algorithms.** The red color means the AD vs. NC classification task, black colors denote AD vs. MCI, and blue colors indicate MCI vs. NC. The y-axis indicates the tractography algorithms and x-axis shows the AUC value. In theory, the higher the AUC value, the better the classification performance. However, if the CI of AUCs has some overlap, we cannot conclude that one algorithm is better than the others, even if the mean AUCs are numerically different. As is evident from the three color bars’ horizontal positions, some classifications are more difficult: AD v. NC is the easiest, and MCI v. NC is the most difficult, perhaps in line with expectation.

**Figure 3** shows the CI for the AUC of nine tractography algorithms for the three diagnostic group discrimination tasks on raw matrices. Three different colors represent three diagnostic tasks. As we can see from **Figure 3**, all AUCs in the same diagnostic task have some level of overlap. This means there is no significant difference among the AUCs of nine tractography algorithm for each diagnostic task. Our results from one-way ANOVA (**Table 3**) are also confirmed this. All three computed values of the *F* statistic in **Table 3** (1.111, 1.348, and 1.945) are less than the critical *F*-value (1.9929) for (8,171) degrees of freedom at α = 0.05, thus we have to accept the null hypothesis. This suggests that there are no significant group differences in the mean AUCs computed from nine tractography algorithms for each diagnostic task using raw matrices as the features.

**Table 3:** One-way ANOVA test on the classification performance of nine tractography algorithms for three diagnostic tasks when using the raw matrices as features.

Diagnostic task	Degrees of freedom	*F*	Sig.
AD vs. NC	Between groups	8	1.111	0.358
	Within groups	171		
AD vs. MCI	Between groups	8	1.348	0.223
	Within groups	171		
MCI vs. NC	Between groups	8	1.945	0.056
	Within groups	171		

The “*F*” column presents computed *F* score and the “Sig.” column gives the *p*-value. Results with Sig. value < 0.05 are treated as nominally significant, so no differences were detectable. “Between Groups” represents sum of the squared deviations from the mean between groups, which captures variability between each group. “Within Groups” represents sum of the squared deviations from the mean within groups, which captures variability within each group. We have nine tractography algorithms, so the number of degrees of freedom for the Between Groups comparison is 9-1 = 8. And since we have 20 splits for each algorithm, the number of degrees of freedom for the Within Groups comparison is 20x9-9 = 171. Since α = 0.05 and the number of degrees of freedom = (8,171), we accept *H_0_* if *F*_8,171_ ≤ 1.9929. All our three *F*-values (1.111, 1.348, and 1.945) are all less than 1.9929, so we accept our H_0_. In other words, there are no significant group differences in these nine tractography algorithm-derived networks using the raw matrices as features.

When using the raw matrix data as features, there is no universally superior method that performs best for all tasks. However, the classification problems do differ in difficulty. As expected, it is easier to distinguish healthy controls from the AD group than from the MCI group. In general, classification accuracy depends on the problem but not strongly on the tractography algorithm.

### Comparing Classifications Based on Thresholded Matrices

As described in Section “Feature Extractions” on the “Global Threshold” and “Individual Binary Threshold,” we tried a range of different threshold values from 0.05 to 0.4, at intervals of 0.05. Although the meaning of the threshold value is slightly different between “Global Threshold” and “Individual Binary Threshold,” they have same threshold value range so we presented both results together to check whether the “threshold value” affects classification performance and whether there are any significant differences among these nine tractography algorithms in terms of classification performance using these thresholded matrices as the features.

**Table 4A** summarizes one-way ANOVA *F*-test results on the AUCs using thresholded matrix features. Our critical *F*-value in the degrees of freedom = (7,152) at α = 0.05 is 2.0703. In **Table 4A**, all computed *F*-values larger than 2.0703 have been marked in red, which means in these seven situations the threshold values do affect the AUCs. To further investigate how threshold values affect AUCs on these seven situations, we did *post hoc* multiple group comparisons and Bonferroni correction was adopted to correct for multiple comparison. After applying Bonferroni correction, the three nominally significant situations (ODF-RK2 using the Global Threshold feature method for AD vs. NC, tensor-TL using the Individual Binary Threshold feature method for AD vs. MCI, and the Hough method using Individual Binary Threshold feature method for MCI vs. NC) no longer showed significant differences for different threshold values. Thus, when using the Global Threshold method to extract features, the classification performance of all tractography algorithms in each of three diagnostic tasks are not affected by the threshold values. This is also true for the task AD vs. NC when using the Individual Binary Threshold feature extraction method. **Table 4B** summarizes *post hoc* multiple comparison results for tensor-RK2 and Hough in task AD vs. MCI as well as Tensor-TL and PICo for task MCI vs. NC using the Individual Binary Threshold as the feature extraction method. Only comparisons with statistical significance between different threshold values are shown in **Table 4B**. Using Individual Binary Threshold feature extraction method, the classification performances in the tasks AD vs. MCI and MCI vs. NC are statistically affected by the threshold values for some tractography algorithms: Tensor-RK2, Hough or Tensor-TL and PICo.

**Table 4A:** One-way ANOVA test on the classification performance across different threshold values for nine tractography algorithms in three diagnostic tasks.

		Degrees of freedom	Global threshold						Individual binary threshold					
			AD vs. NC		AD vs. MCI		MCI vs. NC		AD vs. NC		AD vs. MCI		MCI vs. NC	
			*F*	Sig.	*F*	Sig.	*F*	Sig.	*F*	Sig.	*F*	Sig.	*F*	Sig.
Tensor-FACT	Between groups	7	0.410	0.895	0.578	0.773	1.195	0.309	0.507	0.828	1.391	0.213	0.474	0.852
	Within groups	152												
Tensor-RK2	Between groups	7	0.096	0.998	0.245	0.973	0.924	0.490	1.176	0.320	2.336	0.027	1.020	0.419
	Within groups	152												
Tensor-SL	Between groups	7	0.100	0.998	2.055	0.052	0.188	0.988	1.133	0.346	0.566	0.783	0.970	0.456
	Within groups	152												
Tensor-TL	Between groups	7	0.378	0.914	0.072	0.999	0.525	0.815	0.804	0.585	2.285	0.031	2.474	0.020
	Within groups	152												
ODF-FACT	Between groups	7	0.752	0.628	0.412	0.894	0.645	0.718	0.455	0.865	0.608	0.749	0.250	0.972
	Within groups	152												
ODF-RK2	Between groups	7	2.393	0.024	1.030	0.412	0.302	0.952	1.445	0.191	1.030	0.413	1.062	0.391
	Within groups	152												
Probtrackx	Between groups	7	1.410	0.205	0.481	0.847	0.279	0.962	0.423	0.887	0.727	0.649	0.591	0.763
	Within groups	152												
PICo	Between groups	7	0.138	0.995	0.153	0.993	0.579	0.772	0.572	0.778	0.916	0.496	4.114	0.000
	Within groups	152												
Hough	Between groups	7	0.289	0.957	0.887	0.518	0.506	0.829	0.183	0.988	3.049	0.005	2.538	0.017
	Within groups	152												

The *F* column is the computed* F* statistic and the “Sig.” column shows the *p-*value. Only cells with Sig. value < 0.05 are treated as nominally significant. (If “Sig. = 0.000” is written, this value is less than 0.001). Since we have eight threshold values (0.05-0.4 in intervals of 0.05), the number of degrees of freedom for the Between Groups comparison is 8-1 = 7. Moreover, since we have 20 splits for each threshold value, the number of degrees of freedom for the Within Groups comparison is 20x8-8 = 152. Thus, our critical *F*-value for a number of degrees of freedom = (7,152) at α = 0.05 is 2.0703. Our null hypothesis, *H0*, was that there is no significant difference among different threshold values, so we would only reject *H0* when our computed* F-*value > 2.0703. There were three tests with large* F-*values (>2.0703) but they did not pass the Bonferroni correction in *post hoc* comparisons. These cells included the ODF-RK2 method in the task AD vs. NC using the “Global Threshold,” tensor-TL in task AD vs. MCI using the Individual Binary Threshold, and the Hough method in task MCI vs. NC using Individual Binary Threshold. We treated these three cases as not significant. The rest of the cases with large *F*-values (>2.0703) are marked as red in the table and the corresponding *post hoc* multiple group comparisons in these cases are presented in **(B)**.

**Table 4B:** *Post hoc* comparison results for the Individual Binary Threshold method.

Diagnostic tasks	Tractography algorithm	(I) Threshold	(J) Threshold	Mean difference (I-J)	Sig.	95% confidence interval	
						Lower bound	Upper bound
AD vs. MCI	Tensor-RK2	0.05	0.30	-0.09325	0.023	-0.01800	-0.0065
			0.40	-0.08961	0.035	-0.1763	-0.0029
	Hough	0.05	0.25	-0.10897	0.034	-0.2142	-0.0038
MCI vs. NC	Tensor-TL	0.05	0.35	-0.10285	0.014	0.0109	0.1948
	PICo	0.05	0.15	0.11037	0.028	0.0056	0.2151
			0.35	0.12817	0.004	0.0234	0.2329
			0.40	0.12654	0.005	0.0218	0.2313

The “Sig.” column shows the SPSS adjusted *p*-value and only values below 0.05 are treated as nominally significant (Please refer to the footnote for detailed explanation). Only comparisons that passed Bonferroni correction are shown here. 95% confidence interval is on the mean difference (I-J). Using the Individual Binary Threshold as the feature extraction method, the AUCs from some tractography algorithms may be statistically affected by the threshold values chosen for specific diagnostic tasks.

Now we come to our second hypothesis test in this section, to see whether our classification performances vary among different tractography algorithms, when using thresholded matrices as the feature. However, this is a complex question as there are nine tractography algorithms with eight different threshold values; for each threshold value we conduct 20 different splits of the data, which means there are 20 AUCs for each threshold point for each tractography. To simplify the analysis, we picked the threshold value with the largest average AUC for each tractography algorithm and then conducted a one-way ANOVA on AUCs computed from different tractography algorithm-derived thresholded matrices. Our results (Supplementary Table S2) show that all computed *F* Raw Feature-values are less than the critical *F*-value (1.9929) at the α = 0.05 level with degrees of freedom = (8,171). This does not allow us to reject the null hypothesis (*H0*) that there are no statistical differences among the AUCs computed from these nine tractography algorithm-derived thresholded matrices in each diagnostic task, no matter what the Global Threshold or Individual Binary Threshold.

### Comparing Classification Performance after Using PCA

As described in Section “Feature Extractions” (on PCA), we evaluated a range of choices: from the first 10 principal components (PCs) to the first 150 PCs, computed from all the data. Our first hypothesis test is whether the choice of number of PCs affects how each tractography algorithm performs. The second hypothesis test is whether the choice of tractography algorithm affects the classification performance when using certain number of PCs.

For the first hypothesis test, our null hypothesis (*H0*) is that the choice of the number of PCs will not affect the classification performance. We performed one-way ANOVA on the AUCs from 12 different numbers of PCs and our results (**Table 5A**) show that some tractography algorithms in some diagnostic tasks are statistically affected by the number of PCs when using PCA for feature extraction. Interestingly, for the classification task AD vs. NC, only the tensor-RK2 method was affected while for task AD vs. MCI, most tractography algorithms except ODF-RK2 and PICo were affected by the number of PCs used. *Post hoc* comparisons (**Table 5B**) confirmed this. Moreover, from **Table 5B** we can see that in those tractography algorithms affected by the number of PCs, the AUCs for smaller numbers of PCs are higher than the AUCs in larger number of PCs. For example: for the task AD vs. MCI, the AUC of tensor-FACT using 10 PCs is statistically (by 0.14 units) “higher” than same algorithm using 150 PCs. This trend is consistent for all tractography algorithms listed in **Table 5B**, which suggests that these top PCs have recovered enough information for the classification and more PCs may impair the classification accuracy for these tractography algorithms. Comparisons that did not pass Bonferroni correction are not listed in **Table 5B**, so are no significant differences in classification performance among different choice of PCs. In other words, only tests that passed Bonferroni correction are shown in **Table 5B**.

**Table 5A:** One-way ANOVA test on the classification performances across different numbers of PCs for nine tractography algorithms in three diagnostic tasks.

		Degrees of freedom	AD vs. NC		AD vs. MCI		MCI vs. NC	
			*F*	Sig.	*F*	Sig.	*F*	Sig.
Tensor-FACT	Between groups	11	1.312	0.219	1.912	0.039	0.600	0.828
	Within groups	228						
Tensor-RK2	Between groups	11	3.388	0.000	2.065	0.024	0.299	0.986
	Within groups	228						
Tensor-SL	Between groups	11	0.348	0.973	4.128	0.000	0.826	0.614
	Within groups	228						
Tensor-TL	Between groups	11	0.770	0.670	2.886	0.001	0.649	0.786
	Within groups	228						
ODF-FACT	Between groups	11	0.620	0.811	2.250	0.013	0.331	0.978
	Within groups	228						
ODF-RK2	Between groups	11	0.508	0.897	1.142	0.330	2.083	0.022
	Within groups	228						
Probtrackx	Between groups	11	0.260	0.992	4.908	0.000	2.641	0.003
	Within groups	228						
PICo	Between groups	11	0.053	1.000	0.836	0.604	1.074	0.383
	Within groups	228						
Hough	Between groups	11	0.541	0.874	2.417	0.007	0.653	0.782
	Within groups	228						

Since we have 12 possible numbers of PCs (10∼150), the number of degrees of freedom for the Between Groups comparison is 12-1 = 11. Moreover, since we have 20 splits for each number of PCs, the number of degrees of freedom for the Within Groups comparison is 20 x 12-12 = 228. Thus, the critical *F*-value = 1.8308 at the α = 0.05 level with degrees of freedom = (11,228). Our null hypothesis, *H0*, is that there is no significant difference among different numbers of PCs. We can only reject *H0* when our computed* F-*value > 1.8308, these situations are been marked in red. The corresponding *post hoc* comparison results in these situations are shown in **(B)**.

**Table 5B:** *Post hoc* comparisons results.

Diagnostic tasks	Tractography algorithm	(I) PC number	(J) PC number	Mean difference (I-J)	Sig.	95% confidence interval	
						Lower bound	Upper bound
AD vs. NC	Tensor-RK2	15	75	0.16667	0.003	0.0293	0.3040
			150	0.16759	0.003	0.0302	0.3050
		20	75	0.15370	0.011	0.0163	0.2911
			150	0.15463	0.010	0.0173	0.2920
AD vs. MCI	Tensor-FACT	10	150	0.14378	0.022	0.0092	0.2784
	Tensor-RK2	10	150	0.17437	0.016	0.0150	0.3338
	Tensor-SL	10	40	0.12890	0.017	0.0105	0.2473
			100	0.12099	0.038	0.0026	0.2394
			150	0.19536	0.000	0.0770	0.3137
		15	150	0.17532	0.000	0.0569	0.2937
		20	150	0.12342	0.030	0.0050	0.2418
	Tensor-TL	10	150	0.17099	0.001	0.0383	0.3037
		15	150	0.14747	0.013	0.0148	0.2802
	ODF-FACT	10	150	0.11424	0.019	0.0084	0.2200
	Probtrackx	10	100	0.14219	0.000	0.0519	0.2325
		15	100	0.12236	0.000	0.0320	0.2127
		20	100	0.10876	0.004	0.0184	0.1991
		25	100	0.12500	0.000	00347	0.2153
		30	100	0.13544	0.000	0.0451	0.2258
		35	100	0.13397	0.000	0.0436	0.2243
		40	100	0.10506	0.006	0.0147	0.1954
		45	100	0.09726	0.019	0.0069	0.1876
		50	100	0.09515	0.026	0.0048	0.1855
	Hough	10	150	0.12416	0.008	0.0154	0.2329
		15	150	0.11994	0.014	0.0112	0.2287
MCI vs. NC	ODF-RK2	40	150	0.11920	0.012	0.0125	0.2259
	Probtrackx	10	100	0.12047	0.002	0.0247	0.2162
		15	100	0.09728	0.041	0.0016	0.1930

Only tests that passed Bonferroni correction are shown here. Using PCA as a feature extraction method, the AUCs for some tractography algorithms are statistically affected by the number of PCs for specific diagnostic tasks. Moreover, a smaller number of PCs tends to give better performance (higher AUC) than higher numbers of PCs for these tractography algorithms when using PCA.

Based on this reasoning, we use 10 PCs for each tractography algorithm and conducted one-way ANOVA across the nine tractography algorithms to check this section’s second null hypothesis (*H0*): the classification performances when using PCA from different tractography algorithm show no significant difference for each diagnostic task. Our result (**Tables 6A,B**) shows that for tasks AD vs. NC and MCI vs. NC, we have to reject *H0*, but for AD vs. MCI, we cannot reject *H0*. In both task AD vs. NC and task MCI vs. NC, there exist some comparisons between some tractography algorithms which do pass Bonferroni correction (**Table 6B**) while for task AD vs. MCI, although the *F-*value in one-way ANOVA (**Table 6A**) are larger than the critical *F*-value (= 1.9929 at the α = 0.05 level with the degrees of freedom (8,171)), this does not pass Bonferroni correction, so we can conclude that the classification performances when using PCA from different tractography algorithm have no detectable difference for the task AD vs. MCI. Moreover, for the task MCI vs. NC, Probtrackx performs significantly better than most deterministic tractography approaches (tensor-FACT, RK2, TL and ODF-RK2; **Table 6B**).

**Table 6A:** Statistical analysis results for classification performances from nine tractography algorithms using PCA. **(A)** One-way ANOVA.

Task		Degrees of freedom	*F*	Sig.
AD vs. NC	Between groups	8	3.144	0.002
	Within groups	171		
AD vs. MCI	Between groups	8	2.191	0.030
	Within groups	171		
MCI vs. NC	Between groups	8	2.728	0.007
	Within groups	171		

We have nine tractography algorithms, so the number of degrees of freedom for the Between Groups comparison is 9-1 = 8. And since we have 20 splits for each algorithm, the number of degrees of freedom for the Within Groups comparison is 20x9-9 = 171. Since α = 0.05 and the number of degrees of freedom = (8,171), we accept *H_0_* if *F*_8,171_ ≤ 1.9929. All our three *F*-values (3.144, 2.191, and 2.728) are larger than 1.9929, so we reject our *H_0_*; in other words, there are significant differences in these nine tractography algorithms in classification using PCA to extract features. However, for the task AD vs. MCI, no group comparison passes Bonferroni correction in the *post hoc* tests.

**Table 6B:** *Post hoc* group comparisons.

Task	(I) Tractography algorithm	(J) Tractography algorithm	Mean difference (I-J)	Sig.	95% confidence interval	
					Lower bound	Upper bound
AD vs. NC	Tensor-SL	ODF-FACT	-0.09444	0.006	-0.1741	-0.0148
		ODF-RK2	-0.09028	0.011	-0.1700	-0.0106
CMI vs. NC	Probtrackx	Tensor-FACT	0.10109	0.011	0.0119	0.1903
		Tensor-RK2	0.10091	0.011	0.0117	0.1901
		Tensor-TL	0.09339	0.030	0.0042	0.1826
		ODF-RK2	0.09348	0.030	0.0043	0.1827

The “Sig.” column show the SPSS adjusted *p*-value; only values 0.05 are treated as significant. Only comparisons that passed Bonferroni correction are listed here. For the task AD vs. NC, the classification performance of tensor-SL is significantly poorer than that of ODF-FACT or ODF-RK2. Interestingly, for the task MCI vs. NC, Probtrackx has statistically better performance than the four deterministic tractography algorithms (tensor-FACT, RK2, TL, and ODF-RK2).

### Comparing Classification Performance after Using GLRAM

In this section, we tested another feature extraction (dimension reduction) method, “GLRAM.” Our first hypothesis test is whether the classification performance of each tractography algorithm shows any differences when using GLRAM with different levels of dimension reduction. Our second hypothesis test is whether the classification performance of different tractography algorithms shows any differences when using GLRAM as a feature extraction method.

In GLRAM, an important parameter is to determine the dimension of the reduced matrix; different dimensions for the reduced matrix may lead to different results. For example, a higher dimension for the reduced matrix means less information loss, although it is not clear whether this helps for classification. Thus, we tested a range of reduced dimension parameters, from 5 to 35, at intervals of 5 (in total, seven different dimensions).

**Tables 7A,B** illustrate the influence of the dimension parameter on the classification performance of each tractography algorithm using GLRAM. For one task (AD vs. NC), the tractography algorithms’ performance was not affected by the dimension parameter of GLRAM. In other tasks, only one tractography algorithm’s performance in each task was affected by the choice of the dimension parameter in GLRAM (**Table 7B**). For example, by using Probtrackx in task AD vs. MCI, the lower dimension dataset (5) gave better performance than higher dimension sets (10, 25, 30, and 35). This is evident because the mean differences are all significant and positive; even so, the trend appears to be opposite when using the Tensor-SL approach for the task MCI vs. NC.

**Table 7A:** One-way ANOVA testing for differences in the classification performances across different GLRAM dimension parameters for nine tractography algorithms in three diagnostic tasks.

		Degrees of freedom	AD vs. NC		AD vs. MCI		MCI vs. NC	
			*F*	Sig.	*F*	Sig.	*F*	Sig.
Tensor-FACT	Between groups	6	1.790	0.106	0.662	0.681	1.457	0.198
	Within groups	133						
Tensor-RK2	Between groups	6	1.299	0.262	0.564	0.759	2.438	0.029
	Within groups	133						
Tensor-SL	Between groups	6	1.445	0.202	0.403	0.876	3.010	0.009
	Within groups	133						
Tensor-TL	Between groups	6	2.169	0.049	1.094	0.369	1.590	0.155
	Within groups	133						
ODF-FACT	Between groups	6	0.384	0.888	2.540	0.023	1.398	0.220
	Within groups	133						
ODF-RK2	Between groups	6	1.824	0.099	0.523	0.790	1.930	0.080
	Within groups	133						
Probtrackx	Between groups	6	1.385	0.225	3.800	0.002	0.817	0.559
	Within groups	133						
PICo	Between groups	6	0.178	0.982	0.448	0.845	0.314	0.929
	Within groups	133						
Hough	Between groups	6	0.536	0.780	0.661	0.681	1.083	0.376
	Within groups	133					

Since we have seven possible dimension parameters (5–35), the degrees of freedom for Between Groups is 7-1 = 6. Moreover, since we have 20 splits for each setting of the dimension parameter, the number of degrees of freedom for the Within Groups comparison is 20x7-7 = 133. Thus, the critical* F-*value = 2.1674 at the α = 0.05 level, when the number of degrees of freedom = (6,133). Our *H0* is that there is no significant difference among different dimension parameters. We were able to reject *H0* at the nominal significance level when our computed* F-*value > 2.1674; this included the cases where tensor-TL was used for the task AD vs. NC; ODF-FACT and Probtrackx in task AD vs. MCI; tensor-RK2, SL in task MCI vs. NC. However, three of these cases – including tensor-TL in the task AD vs. NC, ODF-FACT for the task AD vs. MCI, and tensor-RK2 for the task MCI vs. NC – did not pass the Bonferroni correction in the *post hoc* comparisons. There are therefore no significant differences in the classification performance when changing the dimension parameters in these three cases. We marked the other two cases in red and the corresponding *post hoc* results for these situations are shown in **(B)**.

**Table 7B:** *Post hoc* comparisons.

Task	Tractography algorithm	(I) Dimension	(J) Dimension	Mean difference (I-J)	Sig.	95% confidence interval	
						Lower bound	Upper bound
AD vs. MCI	Probtrackx	5	10	0.11181	0.016	0.0111	0.2126
			25	0.10728	0.026	0.0065	0.2080
			30	0.12289	0.005	0.0221	0.2236
			35	0.13449	0.001	0.0337	0.2352
MCI vs. NC	Tensor-SL	5	10	-0.09339	0.045	-0.1857	-0.0011
			35	-0.10498	0.012	-0.1973	-0.0127

The “Sig.” column shows the SPSS adjusted *p*-value; only values 0.05 are treated as significant. Only comparisons that passed Bonferroni correction are listed here. Even for the situations listed in this table, there is no consistent trend in the comparison between higher and lower dimensions. For example in the task of classifying AD vs. MCI, using Probtrackx, the lower dimension setting (5) has better performance than higher dimensional settings (10, 25, 30, and 35), as mean differences are all positive; however, for the task MCI vs. NC, the trend is opposite when using Tensor-SL.

After we studied the effects of GLRAM dimension parameters on each tractography algorithm in terms of its classification performance, we picked up the dimension parameter with the largest average AUC for each tractography algorithm and then conducted one-way ANOVA across nine tractography algorithms to test our null hypothesis (*H0*) that the choice of tractography algorithms does not affect the classification performance. Our result (Supplementary Table S3) is favor of accepting *H0*, in other words, there is no evidence that the choice of tractography algorithms affects the classification performance when using GLRAM as the feature extraction method.

### Comparison of Different Feature Extraction Methods

Finally, we tested whether the classification performance is affected by the choice of the feature extraction methods. For each tractography algorithm, there are five feature extraction choices, which include “Raw feature,” “Global Threshold” using the threshold value with the highest average AUC, “Individual Binary Threshold” using the threshold value with the highest average AUC, “PCA” using 10 PCs, “GLRAM” using the dimension parameter with the highest average AUC. Here our null hypothesis (*H0*) was that there are no significant differences among these five feature extraction methods for any tractography algorithm. So we performed one-way ANOVA on each of nine tractography algorithm among the five feature extraction methods, for each diagnostic task. Our results (**Table 8A**) show that this depends on the diagnostic task as well as the choice of tractography algorithm. For example, the performance of tensor-SL (**Table 8A**) was significantly affected by the choice of feature extraction methods for the task AD vs. NC but not in the other tasks. Some tractography algorithms’ performance was statistically consistent across different feature extraction methods, such as tensor-FACT in all three tasks (**Table 8A**). We also performed *post hoc* tests on the “significant” cases in **Table 8A** and our results (**Table 8B**) showed that after Bonferroni correction, some feature extraction methods perform significantly better than other extraction methods for some tasks, but not in other tasks, so the trend is not consistent. The diagnostic task and the choice of tractography algorithm both affect the results, so there is no universally optimal method.

**Table 8A:** One-way ANOVA on the classification performances across five feature methods for nine tractography algorithms in three diagnostic tasks.

		Degrees of freedom	AD vs. NC		AD vs. MCI		MCI vs. NC	
			*F*	Sig.	*F*	Sig.	*F*	Sig.
Tensor-FACT	Between groups	4	1.491	0.211	1.125	0.349	1.810	0.133
	Within groups	95						
Tensor-RK2	Between groups	4	0.850	0.497	2.029	0.097	1.528	0.200
	Within groups	95						
Tensor-SL	Between groups	4	4.003	0.005	0.196	0.940	0.599	0.664
	Within groups	95						
Tensor-TL	Between groups	4	0.903	0.466	3.246	0.015	1.097	0.363
	Within groups	95						
ODF-FACT	Between groups	4	2.243	0.070	1.622	0.175	1.383	0.246
	Within groups	95						
ODF-RK2	Between groups	4	1.204	0.314	3.745	0.007	2.605	0.041
	Within groups	95						
Probtrackx	Between groups	4	3.498	0.010	0.124	0.974	1.222	0.307
	Within groups	95						
PICo	Between groups	4	0.791	0.534	0.355	0.840	2.793	0.031
	Within groups	95						
Hough	Between groups	4	0.734	0.571	6.051	0.000	0.696	0.597
	Within groups	95						

Since we have five feature extraction methods, the number of degrees of freedom for the Between Groups comparison is 5-1 = 4. Moreover, as we have 20 splits for each setting for the dimension parameter, the number of degrees of freedom for the Within Groups comparison is 20x5-5 = 95. Thus, the critical* F-*value = 2.4675 at α = 0.05 with degrees of freedom = (4,95). Our *H0* is that there is no significant difference among different feature extraction methods; we should therefore only reject *H0* when the computed* F-*value > 2.4675; these nominally “significant” situations are marked in red, except for ODF-RK2 in the task MCI vs. NC as this case did not pass the Bonferroni correction in the *post hoc* group tests. All “significant” cases’ corresponding *post hoc* results are listed in **(B)**.

**Table 8B:** *Post hoc* comparisons.

Diagnostic tasks	Tractography algorithm	(I) Feature extraction method	(J) feature extraction method	Mean difference (I-J)	Sig.	95% confidence interval	
						Lower bound	Upper bound
AD vs. NC	Tensor-SL	Individual binary threshold	PCA	0.09259	0.005	0.0186	0.1666
	Probtrackx	Raw feature	GLRAM	0.05833	0.042	0.0011	0.1155
		Global threshold	GLRAM	0.06296	0.021	0.0058	0.1202
AD vs. MCI	Tensor-TL	Individual binary threshold	Raw feature	0.09900	0.020	0.0096	0.1884
	ODF-RK2	PCA	GLRAM	0.10348	0.007	0.0183	0.1887
	Hough	Individual binary threshold	Raw feature	0.10612	0.007	0.0193	0.1930
			Global threshold	0.08966	0.038	0.0028	0.1765
			GLRAM	0.10232	0.010	0.0155	0.1892
		PCA	Raw feature	0.09768	0.017	0.0108	0.1845
			GLRAM	0.09388	0.025	0.0070	0.1807
MCI vs. NC	PICo	Individual binary threshold	Raw feature	0.09697	0.036	0.0035	0.1904

The “Sig.” column show the SPSS adjusted *p*-value; only values 0.05 are treated as significant. Only comparisons that passed Bonferroni correction are listed here. Although some methods show better performance for some tractography algorithms in some specific tasks, the trend is not consistent, and there is no universally optimal method.

## Discussion

In this study, we have adopted five feature extraction methods on nine whole brain tractography derived brain networks for three diagnostic classification tasks. There are three possible factors affecting the ultimate classification accuracy: the difficulty of the diagnostic task, the feature extraction method and the tractography algorithm.

Our result (**Figure 3**) shows the classification accuracy is strongly correlated with the difficulty of the diagnostic task. The AD vs. NC is the easiest task since there are clear evidences to show that the AD patients have detectable pathological changes (such as short term memory loss, problems with language, disorientation, mood swings, loss of motivation, not managing self care, and behavioral issues) in the brain structures and functions (Burns, 2009; Burns and Iliffe, 2009; Querfurth and LaFerla, 2010; Ballard et al., 2011; Fritze et al., 2011; Todd et al., 2013). While MCI is found to be a transitional stage between normal aging and dementia (Schroeter et al., 2009), the detectable pathological changes of MCI is smaller than AD. Also, there are several subtypes of MCI (e.g., early-MCI and late-MCI; Jessen et al., 2014). So it may increase the complexity to define a simple trend in MCI patient group. Based on our result in **Figure 3**, the difficulty of the diagnostic task has the order: NC vs. MCI > AD vs. MCI > AD vs. NC.

The five feature extraction methods have been widely used in machine learning area (Mocks and Verleger, 1986; Ye, 2005; Yuan and Zou, 2013). Although the “raw features” approach suffers from the “curse of dimensionality” problem, one apparent advantage of this approach is that the method does not require additional computation. For “global threshold,” we need to compute the mean matrix and apply the matrix to each sample, which costs O(NxM) where *M* = 6328. In “individual binary threshold,” we also need to scan through the matrices, the costs of which is still O(NxM). For principle components analysis approach, we need to solve a singular value decomposition problem, which is O(N M^2^) in our case. Given *N* is very small, the complexity can be thought as O(M^2^). GLRAM uses an iterative algorithm, and in each iteration the major computation costs are to solve two top-*k* eigen-decomposition problems, where *k* is the dimension of the reduced matrix. Since the size of the reduce *k* is typically much smaller than D, the operation can be considered as order O(D^2^). Although different algorithms have different computational complexities, and all of them can be efficiently computed in modern computers given the scale of our data. In our experiments, the difference in time costs among these algorithms cannot be noticed. In terms of ultimate classification accuracy, there is no feature extraction method is significantly better than others. However for each feature extraction method, the parameter setting may or may not affect the later result. For example, global threshold or GLRAM is less affected by the parameter selection while the performance of PCA and Individual Binary Threshold indeed change with the parameters.

In this study, nine tractography algorithms were selected from two categories: the deterministic approach and the probabilistic approach. Deterministic tractography recovers fibers emanating from a seed voxel by following the principal direction of the diffusion tensor or the dominant direction of the diffusion ODF. Although the deterministic tractography approach is very efficient, it depends on the choice of initial seed points and can be sensitive to the estimated principal directions. Probabilistic tractography approaches can be computationally more intensive but they are in some cases more robust to partial volume averaging effects and uncertainties in the underlying fiber direction, which are inevitable due to imaging noise. This work is part of a body of work that has examined relative merits of different tractography methods for different tasks and goals. One annual conference, MICCAI, has held annual competitions in recent years. The most recent, in 2014, brought together 15 international tractography teams from leading academic centers. Fillard et al. (2011) evaluated 10 fiber reconstruction methods were evaluated on a specially constructed phantom dataset, with known ground truth on the geometry of the fibers. For medium to low SNR datasets, the authors recommended the use of a prior on the spatial smoothness of either the diffusion model to improve tractography results. Later work on real datasets compared how well different methods could recover the corticospinal tract (CST) from two diffusion imaging data from neurosurgical cases (Neher et al., 2012). Girard et al. (2014) also compared settings in tractography methods (e.g., stopping criteria and seeding techniques) to avoid biases in over-selecting shorter fibers (Girard et al., 2014).

Before we ran this study, we carefully examined the parameter setting for each tractography to make sure the tracked fibers are reasonable. And these parameter settings have been used in lots of other studies in our group. We admitted there are many factors that can affect the performance of each tractography algorithm. All these nine whole brain tractography were computed using LONI Pipeline^8^, which is a 306-node, dual-processor SUN Microsystems V20z cluster. As for the computing time, tensor-based deterministic approaches (tensor-FACT, RK2, SL, and TL) only take less than 5 min and ODF-based deterministic approaches (ODF-FACT, RK2) take about 6∼7 min per brain per CPU. PICo takes about 6∼8 h and Hough takes about 22∼24 h per brain per CPU. And for FSL PROBTRACTX, the prerequisite step BEDPOSTX requires ∼6 h using 110 CPUs per brain and PROBTRACTX requires another 2∼3 h per brain per CPU. So the computation cost for these nine tractography algorithms are Probtrackx > Hough > PICo > ODF-FACT, RK2 > tensor-FACT, RK2, SL, TL. However, our results indicated that although there may have some differences in terms of fiber length or fiber tracking directions for each specific tractography (**Figure 2**), the classification accuracy has no relationship with the computation cost. There were no detectable differences in classification performance among different tractography algorithms when using the raw matrices (**Table 3**), a global or individual binary threshold (Supplementary Table S2) or the GLRAM method (Supplementary Table S3) as feature extraction methods for each of the three diagnostic tasks. However, when using PCA (**Table 6**), for task the AD vs. NC, ODF-based deterministic approaches are better than tensor-based deterministic approach; for the task MCI vs. NC, Probtrackx performed better than deterministic approaches, while for the task AD vs. MCI, there is no significant difference among these nine tractography algorithms. As a general principle, we could expect the probabilistic methods to do better than deterministic methods, and high-order ODF methods should perform better than tensor models, but only in tasks where the additional information is useful. MCI vs. NC is a relatively hard classification task and perhaps best suited to pick up the advantages of more accurate modeling of diffusion and connectivity. In general, the simpler methods already give good performance on the easier classification tasks (e.g., AD vs. NC), while more challenging task tended to benefit from more sophisticated modeling. Moreover, the number of PCs chosen did affect the classification, with a small number of PCs (such as 10) being best in some cases, when adopting PCA as feature extraction method in all three diagnostic tasks (**Tables 5A,B**).

The choice of feature extraction method depends on the problem and the level of sophistication needed for modeling the networks. For tasks that were easier (i.e., when groups differed more), we often failed to detect any practical difference across methods or processing choices. But differences were evident in performance on some of the trickier classification problems. The optimal feature extraction method and tractography algorithm may depend on the diagnostic task. For example, for the somewhat easy classification task of separating AD from NC, individual binary thresholding performed better than PCA for tensor-SL and GLRAM performed worse than using the raw matrices or a global threshold for Probtrackx. This is probably because the AD vs. NC differences are so severe that they can even be picked up by simply binary thresholding on the matrix elements, without requiring more sophisticated data reduction. There were no detectable differences for the rest of seven tractography algorithms in terms of these five feature extraction methods. Similar situations for the tasks AD vs. MCI and MCI vs. NC can be found in **Table 8B**.

Finally, in this study we selected sparse logistic regression classification for performance evaluation and we admitted that the choice of classification algorithm also affects the result. In fact, we have tried different classification algorithms, such as linear support vector machine (SVM), Gaussian SVM or Random Forest, in a separate study. We didn’t include this part here since it will make the content too complex.

## Conclusion

Here, we studied how the choice of tractography method affects our ability to classify different diagnostic groups in aging populations, including AD, based on structural brain networks. Clearly, the diffusion MRI-derived networks would not be the only feature used for diagnostic classification in any realistic setting, but it is important to know which methods perform best (or worst), and with what parameter choices. Most practically, we wanted to know if any methods are best avoided when trying to sensitively detect disease effects on structural brain networks.

Our study had three main findings. First, and as expected, some diagnostic groups were easier to distinguish than others (**Figure 3**). Diagnostic accuracy was higher for AD vs. NC comparisons than for NC vs. MCI comparisons. Differences among algorithms within a task were relatively small compared to the differences in accuracy for different tasks. Secondly, the best tractography algorithm for each diagnostic task differs, but there were no universally optimal methods, or feature extraction methods. Lastly, we tested if any choice of dimensionality reduction method was helpful for classification. With some exceptions, there did not seem to be a universally helpful method (**Tables 8A,B**). The optimal feature extraction method may also depend on the diagnostic task and the choice of the tractography algorithms.

Our study has some limitations. First, it could be argued that the method that best classified patients from controls need not be the most accurate method in the sense of extracting “true” fibers. For instance, a tractography method that fails to extract fibers in certain parts of the brain would not normally be considered the best, even if it gave good classification performance. So an important ancillary study might compare the accuracy of these methods relative to ground truth. A second limitation is that DWI would not normally be the method of choice to classify AD stages; structural atrophy, or amyloid and tau proteins measured in the blood or CSF tends to be favored, because of the known pathology of the disease. Even so, our goal was not to achieve optimal classification, but evaluate tractography features and methods likely to contribute to classification. In the meantime, having a workflow to classify brain networks might offer an additional diagnostic tool for AD and might help other studies unrelated to AD. Also, the following caveats apply. We cannot rule out that the algorithm performance depends on the quality of the data used for testing. The ADNI data is somewhat standard and makes sense to use for evaluation, as it is typical of that collected in DWI studies of clinical cohorts.

Finally, we concede that network analysis is an evolving field. In this study, we chose brain parcellation based on 113 regions. Other atlas and partitions are possible, and it is even possible to adapt the cortical parcellation to achieve better classification. Moreover, slightly changing the parameter setting (such as critical angle) for each tractography method could also affect the performance. To avoid adding too many free parameters to the study, we did not explore these issues here, but such tuning tactics are plausible to improve the discriminatory power of network analysis for any particular tractography or feature set.

## Conflict of Interest Statement

The authors declare that the research was conducted in the absence of any commercial or financial relationships that could be construed as a potential conflict of interest.

## Footnotes

1 For the rest of the paper, we use ‘fiber’ to mean an individual streamline or 3D curve detected by a tractography algorithm. Clearly it would need to be validated whether a streamline represents a true fiber in the brain, but the term ‘fiber’ is less cumbersome so we use it here.

2 http://adni.loni.usc.edu

3 http://adni-info.org/Scientists/Pdfs/ADNI2_Protocol_FINAL_20100917.pdf

4 www.bic.mni.mcgill.ca/software/

5 http://www.fmrib.ox.ac.uk/fsl

6 http://trackvis.org/dtk/

7 http://cmic.cs.ucl.ac.uk/camino/

8 http://pipeline.loni.usc.edu/
