# Supplementary material for: Comparison of nine tractography algorithms for detecting abnormal structural brain networks in Alzheimer’s disease
Source: Front Aging Neurosci. 2015 Apr 14;7:48. doi: 10.3389/fnagi.2015.00048 (PMC4396191; doi:10.3389/fnagi.2015.00048)
Supplement: Supplementary file 1 [file Table_1.DOCX]

**Supplementary Table 1. Names of the 113 ROIs.** Except for the brainstem, each ROI has two numbers, denoting its left (small number) and right (large number) components. The brainstem is not divided into left and right components in the atlas.

| 1 | | Brainstem | 2 | 58 | Thalamus |
| --- | --- | --- | --- | --- | --- |
| 13 | 69 | Inferior_Frontal_Gyrus_Pars_Triangularis | 3 | 59 | Caudate |
| 14 | 70 | Inferior_Frontal_Gyrus_Pars_Opercularis | 4 | 60 | Putamen |
| 17 | 73 | Superior_Temporal_Gyrus_Anterior_Division | 5 | 61 | Pallidum |
| 18 | 74 | Superior_Temporal_Gyrus_Posterior_Division | 6 | 62 | Hippocampus |
| 19 | 75 | Middle_Temporal_Gyrus_Anterior_Division | 7 | 63 | Amygdala |
| 20 | 76 | Middle_Temporal_Gyrus_Posterior_Division | 8 | 64 | Accumbens |
| 21 | 77 | Middle_Temporal_Gyrus_TemporoOccipital_Part | 9 | 65 | Frontal_Pole |
| 22 | 78 | Inferior_Temporal_Gyrus_Anterior_Division | 10 | 66 | Insular_Cortex |
| 23 | 79 | Inferior_Temporal_Gyrus_Posterior_Division | 11 | 67 | Superior_Frontal_Gyrus |
| 24 | 80 | Inferior_Temporal_Gyrus_TemporoOccipital_Part | 12 | 68 | Middle_Frontal_Gyrus |
| 27 | 83 | Supramarginal_Gyrus_Anterior_Division | 15 | 71 | Precentral_Gyrus |
| 28 | 84 | Supramarginal_Gyrus_Posterior_Division | 16 | 72 | Temporal_Pole |
| 30 | 86 | Lateral_Occipital_Cortex_Superior_Division | 25 | 81 | Postcentral_Gyrus |
| 31 | 87 | Lateral_Occipital_Cortex_Inferior_Division | 26 | 82 | Superior_Parietal_Lobule |
| 34 | 90 | Juxtapositional_Lobule_Cortex | 29 | 85 | Angular_Gyrus |
| 35 | 91 | Subcallosal_Cortex | 32 | 88 | IntraCalcarine_Cortex |
| 36 | 92 | ParaCingulate_Gyrus | 33 | 89 | Frontal_Medial_Cortex |
| 37 | 93 | Cingulate_Gyrus_Anterior_Division | 48 | 104 | Occipital_Fusiform_Cortex |
| 38 | 94 | Cingulate_Gyrus_Posterior_Division | 49 | 105 | Frontal_Opercular_Cortex |
| 39 | 95 | PreCuneous_Cortex | 50 | 106 | Central_Opercular_Cortex |
| 40 | 96 | Cuneal_Cortex | 51 | 107 | Parietal_Opercular_Cortex |
| 41 | 97 | Frontal_Orbital_Cortex | 52 | 108 | Planum_Polare |
| 42 | 98 | Parahippocampal_Gyrus_Anterior_Division | 53 | 109 | Heschls_Gyrus |
| 43 | 99 | Parahippocampal_Gyrus_Posterior_Division | 54 | 110 | Planum_Temporale |
| 44 | 100 | Lingual_Gyrus | 55 | 111 | Supracalcarine_Cortex |
| 45 | 101 | Temporal_Fusiform_Cortex_Anterior_Division | 56 | 112 | Occipital_pole |
| 46 | 102 | Temporal_Fusiform_Cortex_Posterior_Division | 57 | 113 | Cerebellum |
| 47 | 103 | Temporal_Occipital_Fusiform_Cortex |  |  |  |
