# Supplementary material for: Comparison of nine tractography algorithms for detecting abnormal structural brain networks in Alzheimer’s disease
Source: Front Aging Neurosci. 2015 Apr 14;7:48. doi: 10.3389/fnagi.2015.00048 (PMC4396191; doi:10.3389/fnagi.2015.00048)
Supplement: Supplementary file 2 [file Table_2.DOCX]

**Supplementary Table 2. One-Way ANOVA on AUCs computed from 9 tractography algorithm-derived thresholded matrices.** The threshold value for each tractography was different and we chose the one with the largest average AUC from 8 possible threshold values (0.05~0.40). Again the degree of freedom for “Between Groups” is 9-1=8 and the degree of freedom for “Within Groups” is 9x20-9=171, so our critical F value at α=0.05 level is 1.9929. Our computed F values in this table are all less than 1.9929, which means there is no evidence to reject the *H0*, in other words, there are no statistical differences among the AUCs from these 9 tractography algorithm-derived thresholded matrices in each diagnostic task, no matter Global Threshold or Individual Binary Threshold.

| Diagnostic Task |  | | Degree of freedom | Global Threshold | | Individual Binary Threshold | |
| --- | --- | --- | --- | --- | --- | --- | --- |
|  |  |  |  | F | Sig. | F | Sig. |
| AD vs NC |  | Between Groups | 8 | 1.296 | .248 | .531 | .832 |
|  |  | Within Groups | 171 |  |  |  |  |
| AD vs MCI |  | Between Groups | 8 | .857 | .554 | 1.583 | .133 |
|  |  | Within Groups | 171 |  |  |  |  |
| MCI vs NC |  | Between Groups | 8 | 1.590 | .131 | 1.906 | .062 |
|  |  | Within Groups | 171 |  |  |  |  |
