# Supplementary material for: Comparison of nine tractography algorithms for detecting abnormal structural brain networks in Alzheimer’s disease
Source: Front Aging Neurosci. 2015 Apr 14;7:48. doi: 10.3389/fnagi.2015.00048 (PMC4396191; doi:10.3389/fnagi.2015.00048)
Supplement: Supplementary file 3 [file Table_3.DOCX]

**Supplementary Table 3. One-Way ANOVA on AUCs computed from 9 tractography algorithm when using GLRAM as feature extraction method.** The GLRAM dimension parameter for each tractography was different and we chose the one with the largest average AUC from 7 possible threshold values (5~35). Again the degree of freedom for “Between Groups” is 9-1=8 and the degree of freedom for “Within Groups” is 9x20-9=171, so our critical F value at α=0.05 level is 1.9929. Our computed F values in this table are all less than 1.9929, which means there is no evidence to reject the *H0*, in other words, there are no statistical differences among the AUCs from these 9 tractography algorithm when using GLRAM in each diagnostic task.

| Diagnostic Task |  | | Degree of freedom | F | Sig. |
| --- | --- | --- | --- | --- | --- |
| AD vs NC |  | Between Groups | 8 | .545 | .822 |
|  |  | Within Groups | 171 |  |  |
| AD vs MCI |  | Between Groups | 8 | .909 | .511 |
|  |  | Within Groups | 171 |  |  |
| MCI vs NC |  | Between Groups | 8 | 1.938 | .057 |
|  |  | Within Groups | 171 |  |  |
